# Identification of Target Genes of the bZIP Transcription Factor OsTGAP1, Whose Overexpression Causes Elicitor-Induced Hyperaccumulation of Diterpenoid Phytoalexins in Rice Cells

**DOI:** 10.1371/journal.pone.0105823

**Published:** 2014-08-26

**Authors:** Koji Miyamoto, Takashi Matsumoto, Atsushi Okada, Kohei Komiyama, Tetsuya Chujo, Hirofumi Yoshikawa, Hideaki Nojiri, Hisakazu Yamane, Kazunori Okada

**Affiliations:** 1 Department of Biosciences, Teikyo University, Utsunomiya, Tochigi, Japan; 2 Biotechnology Research Center, The University of Tokyo, Bunkyo-ku, Tokyo, Japan; 3 Genome Research Center, NODAI Research Institute, Tokyo University of Agriculture, Setagaya-ku, Tokyo, Japan; 4 Department of Bioscience, Tokyo University of Agriculture, Setagaya-ku, Tokyo, Japan; University of Nebraska Medical Center, United States of America

## Abstract

Phytoalexins are specialised antimicrobial metabolites that are produced by plants in response to pathogen attack. Momilactones and phytocassanes are the major diterpenoid phytoalexins in rice and are synthesised from geranylgeranyl diphosphate, which is derived from the methylerythritol phosphate (MEP) pathway. The hyperaccumulation of momilactones and phytocassanes due to the hyperinductive expression of the relevant biosynthetic genes and the MEP pathway gene *OsDXS3* in OsTGAP1-overexpressing (OsTGAP1ox) rice cells has previously been shown to be stimulated by the chitin oligosaccharide elicitor. In this study, to clarify the mechanisms of the elicitor-stimulated coordinated hyperinduction of these phytoalexin biosynthetic genes in OsTGAP1ox cells, transcriptome analysis and chromatin immunoprecipitation with next-generation sequencing were performed, resulting in the identification of 122 OsTGAP1 target genes. Transcriptome analysis revealed that nearly all of the momilactone and phytocassane biosynthetic genes, which are clustered on chromosomes 4 and 2, respectively, and the MEP pathway genes were hyperinductively expressed in the elicitor-stimulated OsTGAP1ox cells. Unexpectedly, none of the clustered genes was included among the OsTGAP1 target genes, suggesting that OsTGAP1 did not directly regulate the expression of these biosynthetic genes through binding to each promoter region. Interestingly, however, several OsTGAP1-binding regions were found in the intergenic regions among and near the cluster regions. Concerning the MEP pathway genes, only *OsDXS3*, which encodes a key enzyme of the MEP pathway, possessed an OsTGAP1-binding region in its upstream region. A subsequent transactivation assay further confirmed the direct regulation of *OsDXS3* expression by OsTGAP1, but other MEP pathway genes were not included among the OsTGAP1 target genes. Collectively, these results suggest that OsTGAP1 participates in the enhanced accumulation of diterpenoid phytoalexins, primarily through mechanisms other than the direct transcriptional regulation of the genes involved in the biosynthetic pathway of these phytoalexins.

## Introduction

Phytoalexins are specialised antimicrobial metabolites that are produced by plants in response to pathogen attack [1]. In rice, momilactones and phytocassanes are recognised as the major diterpenoid phytoalexins [2]–[5].

In plants, isopentenyl diphosphate and dimethylallyl diphosphate, which are the basic C5 precursors for terpenoid biosynthesis, are produced by two distinct pathways: the mevalonate pathway and methylerythritol phosphate (MEP) pathway [6]. In suspension-cultured rice cells, the MEP pathway genes exhibit elicitor-induced expression, and the MEP pathway is required for the production of sufficient amounts of diterpenoid phytoalexins [7]. However, the transcriptional regulatory mechanisms of the MEP pathway genes remain unknown.

In the biosynthesis of diterpenoid phytoalexins, the common precursor geranylgeranyl diphosphate is sequentially cyclised by OsCPS2, OsCPS4, OsKSL7, and OsKSL4 into two distinct diterpene hydrocarbons: *ent*-cassa-12,15-diene and 9βH-pimara-7,15-diene [8]–[10]. For momilactone biosynthesis, two P450 monooxygenases (CYP99A2 and CYP99A3) and a dehydrogenase (OsMAS) are involved in the downstream oxidation of 9βH-pimara-7,15-diene [11], [12]. For phytocassane biosynthesis, four P450 monooxygenases (CYP71Z7, CYP76M7, CYP76M8, and CYP701A8/OsKOL4) are involved in the oxidation of *ent*-cassa-12,15-diene [13]–[16]. The momilactone and phytocassane biosynthetic genes are localized in narrow regions of chromosomes 4 and 2, respectively, creating functional gene clusters [11], [13]. These biosynthetic genes exhibit the temporally coordinated expression of mRNAs after treatment with a biotic elicitor in suspension-cultured rice cells [7], [11].

The basic leucine zipper (bZIP) transcription factor OsTGAP1 has been shown to be involved in the regulation of the production of momilactones and phytocassanes. OsTGAP1-overexpressing (OsTGAP1ox) rice cells exhibit the hyperaccumulation of momilactones and phytocassanes as well as the enhanced expression of all momilactone biosynthetic genes, the phytocassane biosynthetic gene *OsKSL7*, and the MEP pathway gene *OsDXS3*, upon treatment with an elicitor [17]. However, the details of the regulation of these genes by OsTGAP1 remain unknown.

In *Arabidopsis thaliana*, 75 members of the bZIP transcription factor family have been identified and classified into ten groups (group A–I and S) based on sequence similarity among their basic regions and the presence of additional conserved motifs [18]. TGA factors (AtTGA1–7), which belong to the group D bZIP transcription factors, regulate pathogenesis-related genes such as *PR-1* through binding to the TGACG-motif (*as-1*-like element, TGACG[T/G]) on the promoter region and mediate salicylic acid–induced defence responses [19]. The transcriptional regulatory mechanism of *PR-1* has been well studied [20]. However, knowledge regarding the genome-wide binding regions of these TGA factors is limited; only AtTGA2 binding regions have been identified by chromatin immunoprecipitation (ChIP) with tiling arrays containing probes representing the 2-kbp upstream regions of *Arabidopsis* genes [21]. In rice, 89 members of the bZIP transcription factor family have been identified, among which 14 share conserved motifs with the *Arabidopsis* group D bZIP transcription factors [22]. However, the biological functions of only four TGA factors, including OsTGAP1, have been determined. Three TGA factors in rice, viz. rTGA2.1, rTGA2.2, and rTGA2.3, are involved in the regulation of defence responses against *Magnaporthe oryzae* and the rice bacterial blight *Xanthomonas oryzae* pv. *oryzae* [23]–[26]. However, the target genes of these three TGA factors remain unknown.

In the present study, transcriptome analysis and ChIP analysis with next-generation sequencing (ChIP-seq) using untreated and elicitor-treated OsTGAP1ox cells were performed, resulting in the identification of OsTGAP1 target genes. Interestingly, the clustered diterpenoid phytoalexin biosynthetic genes were not included among the OsTGAP1 target genes. However, it should be noted that several OsTGAP1-binding regions were found in the intergenic regions among and near the cluster regions. Concerning the MEP pathway genes, it was found that only *OsDXS3* possessed an OsTGAP1-binding region in its upstream region and that OsTGAP1 directly regulated *OsDXS3* expression, while other MEP pathway genes were not included among the OsTGAP1 target genes. Possible mechanisms by which OsTGAP1 may regulate the production of diterpenoid phytoalexins are also discussed.

## Materials and Methods

### Plants, chemical treatment, and rice transformation

*Oryza sativa* L. ‘Nipponbare’ was used as the wild-type strain. Suspension-cultured rice cells were maintained as described in a previous paper [8]. *N*-acetylchitooctaose was prepared and used to treat the rice cells as a chitin oligosaccharide elicitor, as described previously [17], [27]. Rice transformation was performed as described previously [17].

### Plasmid construction

The *Zea mays* polyubiquitin promoter was amplified by PCR using the primers UBQp attB4 F and UBQp attB1 R from pANDA [28] and cloned into pDONRP4-P1R (Invitrogen, CA, USA), resulting in pDONR-UBQp. The polyubiquitin promoter and OsTGAP1 open reading frame (ORF) were then cloned into R4pGWB501 [29] from pDONR-UBQp and pENTR-TGA [17] using LR Clonase II Plus Enzyme mix (Invitrogen). The resulting plasmid was designated as R4pGWB-UBQp-TGA and used for rice transformation.

The 2-kbp upstream region of *OsDXS3* was amplified by PCR using the primers DXS3p 2k F and DXS3p R and cloned into the *Kpn*I and *Hin*dIII sites of the pGL3-Basic vector (Promega, WI, USA), resulting in pGL3-DXS3p-2k. Next, three mutated constructs were generated: one of the two TGACGT sequences was mutated in m1 and m2, while both TGACGT sequences were mutated in m3. The TGACGT sequences in the *OsDXS3* promoter were mutated by PCR using the following primer pairs: DXS3p-m1-F and DXS3p-m1-R, DXS3p-m2-F and DXS3p-m2-R, and DXS3p-m3-F and DXS3p-m3-R. Each mutated *OsDXS3* promoter was then amplified by PCR using the primers DXS3p 2k F and DXS3p R. The amplified DNA fragments were cloned into the *Kpn*I and *Hin*dIII sites of the pGL3-Basic vector and then sequenced, resulting in pGL3-DXS3p-2k-m1, pGL3-DXS3p-2k-m2, and pGL3-DXS3p-2k-m3.

Fragments of the *OsDXS3* promoter (250 bp and 240 bp) were amplified by PCR using the primers DXS3p 250 F, DXS3p 240 F, and DXS3p R from pGL3-DXS3p-2k. A 250-bp fragment of the *OsDXS3* promoter with mutated TGACGT sequences was also amplified by PCR using the primers DXS3p 250 m1 F, DXS3p 250 m2 F, DXS3p 250 m3 F, and DXS3p R. These amplified DNA fragments were cloned into the *Bgl*II and *Hin*dIII sites of the pGL3-Basic vector and sequenced. The resulting plasmids were designated as pGL3-DXS3p-250, pGL3-DXS3p-240, pGL3-DXS3p-250-m1, pGL3-DXS3p-250-m2, and pGL3-DXS3p-250-m3.

The OsTGAP1 ORF was cloned into pUbi_RfA_Tnos [30] from pENTR-TGA using LR Clonase II Enzyme mix (Invitrogen). The resultant plasmid was designated pUbi_TGA_Tnos and used as the effector plasmid.

A summary of the plasmids used in this study is provided in Table S1, and the sequences of PCR primers used for plasmid construction are provided in Table S2.

### Antibody generation and purification

An OsTGAP1-specific antibody was generated by immunizing rabbits with the keyhole limpet hemocyanin-coupled synthetic peptide MELYPGYLEDHFNIHK corresponding to the N-terminal peptide sequence (residues 1–16) of OsTGAP1. Cys residues were added to the N-terminus of the peptides to ensure efficient coupling to the keyhole limpet hemocyanin carrier protein. The OsTGAP1-specific antibody was further purified using an antigen affinity column.

### Nuclear extraction

Rice cells (approximately 3 g) were ground and suspended in 30 ml of 70% glycerol buffer (20 mM Hepes/NaOH, pH 7.4, 5 mM MgCl_2_, 5 mM KCl, 50 mM saccharose, 70% [v/v] glycerol, 1 mM DTT, and 200 mM phenylmethylsulfonyl fluoride) and incubated for 20 min at 4°C under mild shaking. After the filtration of the slurry through a quadrilayer of Miracloth (Merck, UK), the nuclei were collected by centrifugation (4,000×*g*, 60 min, 4°C) and washed in 10% glycerol buffer (20 mM Hepes/NaOH, pH 7.4, 5 mM MgCl_2_, 5 mM KCl, 50 mM saccharose, 10% [v/v] glycerol, 1 mM DTT, and 200 mM phenylmethylsulfonyl fluoride) by gentle resuspension followed by centrifugation (4,000×*g*, 20 min, 4°C). The nuclei were resuspended in 500 µl of TE buffer (pH 8.0) and sonicated for 1 min with a Sonifier 250D (Branson, CT, USA) at output setting 1 with a pulse of 1 s and duty cycle of 50%. The sonicated nuclei were cleared by centrifugation (20,000×*g*, 15 min, 4°C) followed by ultracentrifugation (100,000×*g*, 60 min, 4°C). The supernatant was then subjected to immunoprecipitation (IP). For protein gel blot analysis, acetone precipitation (95% [v/v] acetone, −20°C, overnight) was performed. The precipitated protein was suspended in Laemmli SDS sample buffer and boiled.

### Conjugation of IgG to Dynabeads Protein G

Conjugation of the anti-OsTGAP1 antibody or normal rabbit IgG to Dynabeads Protein G (Invitrogen) was performed using bis[sulfosuccinimidyl] suberate (Thermo Fisher, MA, USA) as the cross-linking reagent according to the manufacturer’s protocol.

### Immunoprecipitation

NaCl and NP-40 were added to the nuclear protein at final concentrations of 100 mM and 0.4% (v/v), respectively. Next, 50 µl of Dynabeads Protein G washed with IP buffer (10 mM Tris-HCl [pH 8.0], 1 mM EDTA, 100 mM NaCl, 0.4% [v/v] NP-40) was added to the nuclear protein. After incubation at 4°C for 60 min with rotation, the Dynabeads Protein G was removed. A small portion of precleared nuclear protein was collected as an ‘Input’ control. The ‘Input’ control was mixed with Laemmli SDS sample buffer and boiled. The precleared nuclear protein was divided into two aliquots. Dynabeads Protein G cross-linked normal IgG was added to one sample, and Dynabeads Protein G cross-linked anti-OsTGAP1 antibody was added to the other sample. After overnight incubation at 4°C with rotation, the Dynabeads Protein G was collected, and a flow-through sample was then harvested. The Dynabeads Protein G was washed five times in 1 ml of IP buffer and resuspended in 20 µl of 0.2 M glycine-HCl (pH 2.5). After incubation at room temperature for 5 min, the eluted protein was collected. This elution step was performed twice. The eluted protein was neutralized by adding 5 µl of 1 M Tris (pH 11). The resulting sample was then subjected to acetone precipitation. The precipitated protein was suspended in Laemmli SDS sample buffer and boiled.

### Protein gel blot analysis

The boiled samples were subjected to SDS-PAGE on 8% (w/v) polyacrylamide gels and then transferred to Amersham Hybond ECL Nitrocellulose Membranes (GE Healthcare, UK). OsTGAP1 was detected using the anti-OsTGAP1 antibody (dilution 1∶2,500 [v/v]) as the primary antibody and the ECL anti-rabbit IgG horseradish peroxidase–linked species-specific whole antibody (dilution 1∶25,000 [v/v]) (GE Healthcare) as the secondary antibody. Chemiluminescent detection was conducted using the Immobilon Western Chemiluminescent HRP Substrate (Millipore, MA, USA).

### Formaldehyde fixation

Formaldehyde was added to the liquid medium of cultured rice cells at a final concentration of 1% (v/v). After incubation at 25°C for 7 min, 1 M glycine was added to a final concentration of 100 mM to stop fixation. After incubation at 25°C for 5 min, the rice cells were collected by filtration using a Buechner funnel and then flash-frozen in liquid N_2_.

### Chromatin immunoprecipitation

Formaldehyde cross-linked nuclei were extracted from fixed OsTGAP1ox rice cells (6 g) as described in the ‘Nuclear extraction’ section above. After the nuclei were resuspended in 500 µl of TE buffer (pH 8.0), their chromatin was sheared by repetitive sonication with a Bioruptor UCW-201 (Tosho Denki, Japan; power 5, ON time 30 s, OFF time 60 s, 30 cycles). The sonicated chromatin was cleared by centrifugation (20,000×*g*, 15 min, 4°C) followed by ultracentrifugation (100,000×*g*, 60 min, 4°C). NaCl and NP-40 were then added at final concentrations of 100 mM and 0.4% (v/v), respectively, and IP buffer was added to the sonicated chromatin to a final volume of 2.5 ml. Then, 250 µl of Dynabeads Protein G washed with IP buffer was added to the sonicated chromatin. After incubation at 4°C for 60 min with rotation, the Dynabeads Protein G was removed, and 500 µl of precleared sample was collected as an ‘Input’ control. The precleared nuclear protein was then divided into two aliquots. Rabbit normal IgG (2.5 µg) was added to one sample, and 2.5 µg of the anti-OsTGAP1 antibody was added to the other. After overnight incubation at 4°C with rotation, 25 µl of Dynabeads Protein G washed with IP buffer was added. After incubation at 4°C for 60 min with rotation, the Dynabeads Protein G was washed seven times in 10 ml of IP buffer. After the wash, the Dynabeads Protein G was resuspended in 500 µl of TE buffer (pH 8.0), and 20 µl of 5 M NaCl were added. The Dynabeads Protein G was then incubated at 65°C overnight. The ‘Input’ control was also amended with 10 µl of 5 M NaCl and incubated at 65°C overnight. The samples were treated with RNase and Proteinase K, followed by phenol-chloroform extraction and isopropanol precipitation. The precipitated DNA was then dissolved in TE buffer (pH 8.0).

### Next-generation sequencing

The construction of DNA libraries was performed using a Paired-End DNA Sample Prep Kit (Illumina, CA, USA) and a Multiplexing Sample Preparation Oligonucleotide Kit (Illumina) according to the manufacturer’s instructions. In this step, ∼350-bp fragments were collected from each sample using E-Gel SizeSelect 2% (Invitrogen). The constructed libraries were subsequently subjected to deep sequencing using a Genome Analyzer II (Illumina). These reads were mapped to the rice genome with the Burrows-Wheeler Aligner (BWA) software package [31] using the International Rice Genome Sequencing Project genome sequence (build 5) from the Rice Annotation Project Database (RAP-DB: http://rapdb.dna.affrc.go.jp) as the reference genome sequence. The OsTGAP1-binding regions in each sample were then detected using Partek Genomics Suite (ver. 6.5; http://www.partek.com/, Partek Software, MO, USA) according to the following thresholds: window size = 100, peak cut-off false discovery rate (FDR) <0.001, strand separation FDR<0.05, and significant enrichment in ChIP DNA compared to the ‘Input’ control (FDR<0.05). The sequence data were deposited in the DDBJ Sequence Read Archive (http://trace.ddbj.nig.ac.jp/dra/index.html; ID: DRA001274).

### DNA microarray analysis

Wild-type (WT) and OsTGAP1ox rice cells were treated with a chitin oligosaccharide elicitor. Total RNA was then isolated from a small portion of the rice cells using an RNeasy Plant Mini Kit (Qiagen, Germany) at 0, 6, and 24 h after the elicitor treatment and labelled with Cyanine 3 dye (Cy3) or Cyanine 5 dye (Cy5). Aliquots of Cy3-labelled cRNA and Cy5-labelled cRNA (825 ng each) were used for hybridization in a 60-mer rice oligo microarray with 44 k features (Agilent Technologies, CA, USA). Two series of microarray analyses were performed as follow. First, to investigate elicitor responsiveness, a time-course analysis of the elicitor treatment of WT cells was performed. The Cy3-labelled cRNA probe from the 0 h time point of the WT cells was used as a reference, and the Cy5-labelled cRNA probes from the 6 h and 24 h time point samples were compared against the 0 h reference. Second, comparison analysis was performed between the WT and OsTGAP1ox cells. The Cy3-labelled cRNA probes from the WT cells (0, 6, and 24 h after elicitor treatment) were used as references, and the Cy5-labelled cRNA probes from the OsTGAP1ox cells (0, 6, and 24 h after elicitor treatment) were compared against the references at each time point. The glass slides were scanned using a microarray scanner (G2565, Agilent), and the resulting output files were imported into Feature Extraction software (ver. 11; Agilent). These data were deposited into the Gene Expression Omnibus of the NCBI (http://www.ncbi.nlm.nih.gov/geo/; ID: GSE53414 and GSE53417). Statistical analyses were performed using Partek Genomics Suite. The expression levels of the OsTGAP1 target genes at each time point were calculated from fold changes relative to those of the WT cells 0 h after elicitor treatment, and the calculated data were imported into MultiExperiment Viewer (MeV v4.6, http://www.tm4.org/mev/) for cluster analysis. Hierarchical cluster analysis based on the average linkage and cosine correlation was used to cluster the genes on the *y*-axis using MeV.

### ChIP-PCR

Quantitative PCR was performed using Power SYBR Green PCR Master Mix (Applied Biosystems, CA, USA) on an ABI PRISM 7300 Real-Time PCR System (Applied Biosystems). The DNA amount was determined by generating standard curves using a series of known concentrations of the target sequence. The ratio of DNA present in the ChIP DNA to that in the ‘Input’ control was calculated. The primers DXS3p TGACGT F and DXS3p TGACGT R were used for the *OsDXS3* promoter region.

### Gel mobility shift assay (GMSA)

The DNA probe was amplified by PCR from pGL3-DXS3p-2k using the primers DXS3p TGACGT F and DXS3p TGACGT R. Mutated probes in the TGACGT sequence (m1, m2, and m3) were also amplified by PCR from pGL3-DXS3p-2k-m1, pGL3-DXS3p-2k-m2, and pGL3-DXS3p-2k-m3, respectively, using the same primers. These amplified probes were end-labelled with ^32^P by T4 polynucleotide kinase (Takara Bio, Japan). Recombinant N-terminal glutathione *S*-transferase (GST)-fused OsTGAP1 (GST-OsTGAP1) and GST protein were expressed in *E. coli* Rosetta 2 (DE3) harbouring pDEST15-TGA [17] or pGEX-6p2 (GE Healthcare) and purified using Glutathione Sepharose 4B (GE Healthcare). The reaction mixture comprised 20 mM Hepes (pH 7.6), 1 mM EDTA, 10 mM (NH_4_)_2_SO_4_, 1 mM DTT, 0.2% (v/v) Tween 20, 30 mM KCl, 0.5 pmol of recombinant GST or GST-OsTGAP1, and 0.05 pmol of the probe in a final volume of 20 µl. The above mixed samples were incubated for 20 min at room temperature, and then 5 µl of loading buffer (0.25×TBE buffer, 40% [w/v] glycerol, 0.2% [w/v] bromophenol blue) was added. The samples were separated on a 4% polyacrylamide gel in 0.5×TBE at room temperature, and the bands were visualized by autoradiography.

### Transactivation assay

pUbi_GUS_Tnos [30] and pUbi_OsTGAP1_Tnos, in which β-*glucuronidase* (*GUS*) and *OsTGAP1*, respectively, are under the control of the *polyubiquitin* promoter, were used as the effector plasmids. pGL3-DXS3p-2k, pGL3-DXS3p-250, pGL3-DXS3p-240, pGL3-DXS3p-250-m1, pGL3-DXS3p-250-m2, and pGL3-DXS3p-250-m3, in which the *OsDXS3* promoter was fused to the firefly luciferase (*FLUC*) gene, were used as the reporter plasmids. The plasmid pPTRL [32], which contains the *Renilla luciferase* (*RLUC*) gene under the control of the cauliflower mosaic virus (CaMV) *35S* promoter, was used as an internal control. Particle bombardment of rice cells was conducted as previously described [17]. In the cotransfection assays, 0.1 µg or 1 µg of the effector plasmid, 1.0 µg of the reporter plasmid, and 0.5 µg of pPTRL were used for each bombardment. Luciferase (LUC) assays were performed as previously described [17]. The ratio of LUC activity (FLUC/RLUC) was calculated to normalize the values after each assay.

## Results

### Genome-wide identification of *in vivo* OsTGAP1-binding regions

To locate *in vivo* OsTGAP1-binding regions, ChIP-seq analysis was performed. An antipeptide antibody directed against the N-terminal peptide sequence (residues 1–16) of OsTGAP1 was generated, as this sequence has high immunogenicity and low similarity to other proteins in rice. To assess the specificity of the generated anti-OsTGAP1 antibody, protein gel blot analysis of the nuclear extracts from the WT and OsTGAP1ox cells was performed. A single band (∼45 kD) was detected from the nuclear extract of the WT cells, and this band was strengthened in the nuclear extract of the OsTGAP1ox cells (Figure S1A). IP, using the anti-OsTGAP1 antibody, was also performed from the nuclear extracts of the WT cells. When rabbit normal IgG was used for IP, no bands were detected by protein gel blot analysis using the anti-OsTGAP1 antibody. In contrast, a single band thought to represent OsTGAP1 was detected after IP by the anti-OsTGAP1 antibody (Figure S1B). These results indicated that the anti-OsTGAP1 antibody generated in this study had sufficient specificity.

The OsTGAP1ox cells were treated with or without the chitin elicitor for 6 h and then collected for ChIP-seq analysis using the anti-OsTGAP1 antibody. Two biological replicates were performed for each sample. DNA libraries of ChIP DNA from the untreated and elicitor-treated samples were then generated. DNA libraries of the ‘Input’ controls were also generated from each sample. These libraries were then subjected to next-generation sequencing using a Genome Analyzer II (Illumina). A total of 4.5 to 10 million reads (100 bp per read) were obtained from each library. These reads were mapped to the rice genome by BWA [31]. OsTGAP1-binding regions were then detected in each sample. Two biologically independent ChIP-Seq experiments were compared, and the regions that showed enrichment in both datasets were identified as OsTGAP1-binding regions. The results identified 2,763 and 2,777 binding regions from the untreated and elicitor-treated samples, respectively (Table S3, Table S4). A comparison of the binding regions in the two conditions revealed that a common set of 2,003 regions (approximately 70%) were bound by OsTGAP1 both with and without elicitation.

**Figure 1:**
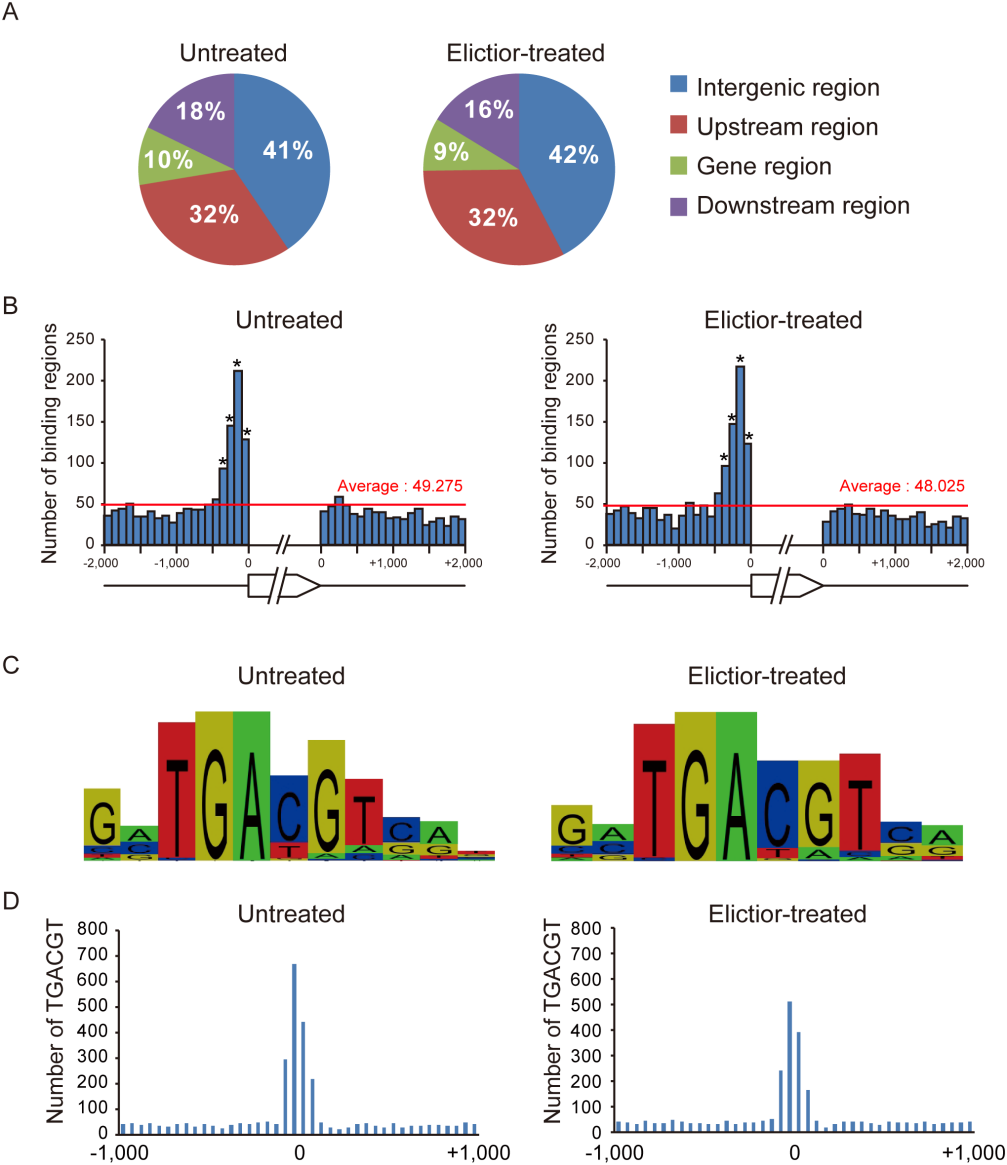
Overview of the results of ChIP-seq. (A) Distribution of OsTGAP1-binding regions in the rice genome. The upstream region includes the binding regions within 2 kbp of the transcription start site. The gene region includes the binding regions that are located between the transcription start site and the transcription termination site. The downstream region includes the binding regions within 2 kbp of the transcription termination site. The remaining binding regions were assigned to intergenic regions. (B) Distribution of OsTGAP1-binding regions in the upstream and downstream regions. Red lines indicate the average of number of OsTGAP1-binding regions. Statistical analysis was performed for each 100-bp region, and significantly enriched regions were indicated by asterisks (*P*<0.01 the binomial test and the Bonferroni correction). (C) Overrepresented motifs in the OsTGAP1-binding regions analysed in the untreated and elicitor-treated conditions using Partek Genomics Suite based on Gibbs motif sampler [37]. (D) Distribution of TGACGT sequences around the OsTGAP1-binding regions.

Subsequent analysis revealed that among all of these OsTGAP1-binding regions, 32% (i.e. 880 and 901 regions) were assigned to the upstream regions of particular rice genes (within 2 kbp of the transcription start site) in the untreated and elicitor-treated conditions, respectively; moreover, 10% and 9% (i.e. 272 and 248 regions) were assigned to the gene regions of particular rice genes (from the transcription start site to the transcription termination site), and 18% and 16% (i.e. 491 and 452 regions) were assigned to the downstream regions of particular rice genes (within 2 kbp of the transcription termination site) in the untreated and elicitor-treated conditions, respectively. Intriguingly, the remaining 41% and 42% of the regions (i.e. 1,120 and 1,176) were assigned to the intergenic regions in the untreated and elicitor-treated conditions, respectively. This proportion is larger than those of the other plant transcription factors (HY5, PIL5, AGL15, and BZR1) whose genome-wide binding regions have previously been reported [33]–[36]. These results are summarized in Figure 1A. The locations of the OsTGAP1-binding regions assigned to the upstream or downstream regions of particular genes were significantly concentrated within 400-bp of the transcription start sites (Figure 1B). This distribution pattern implies that OsTGAP1-binding regions located within 400-bp of the transcription start sites has the significant role on the transcriptional regulation of downstream genes. Therefore, this study focussed on the genes that possessed an OsTGAP1-binding region within the 400-bp upstream region in either the untreated or elicitor-treated condition. A total of 693 genes were identified as potential OsTGAP1 target genes (Table S5).

### Analysis of OsTGAP1-binding motifs

Overrepresented motifs in the OsTGAP1-binding regions were analysed using Partek Genomics Suite based on Gibbs motif sampler [37]. The TGACGT sequence was found in both the untreated and elicitor-treated condition (Figure 1C). These results are consistent with those of a previous report, which observed that OsTGAP1 binds to the TGACGT sequence *in* *vitro* [17]. We also analyzed whether TGACGT sequences are overrepresented in the OsTGAP1-binding regions compared to the rest of genome. As a result, TGACGT sequences are enriched approximately 8-fold in the OsTGAP1-binding regions compared to the rest of genome, although known binding motifs of other transcription factors were not enriched. (Figure S2). Furthermore, analysis of the distribution pattern of the TGACGT sequence around the OsTGAP1-binding regions (−1,000 to+1,000 bp of each predicted binding region) revealed that the occurrence of the TGACGT sequence peaks at the predicted OsTGAP1-binding regions in both the untreated and elicitor-treated condition (Figure 1D).

### Identification of OsTGAP1 target genes

To identify the genes regulated by OsTGAP1, a genome-wide DNA microarray analysis was performed using the WT and OsTGAP1ox cells, and gene expression was compared between these cells at 0, 6, and 24 h after the chitin elicitor treatment. Four biological replicates were used for each time point. Statistical analysis was performed using ANOVA-FDR (*q* value≤0.05), and genes with changes in expression were identified as those that experienced a two-fold increase or decrease of expression level in the OsTGAP1ox cells at least one time point compared to the expression levels in the WT cells. Based on this criterion, 1,352 genes, 1,539 genes, and 1,267 genes were upregulated in the OsTGAP1ox cells at 0, 6, and 24 h after the elicitor treatment, respectively. By combining these three groups, 2,268 genes were identified that were upregulated in the OsTGAP1ox cells at least one time points (Figure S3A, Table S6). Conversely, 1,278 genes, 1,387 genes, and 1,624 genes were downregulated in the OsTGAP1ox cells at 0, 6, and 24 h after the elicitor treatment, respectively. By combining these three groups, 2,276 genes were identified that were downregulated in the OsTGAP1ox cells at least one time points (Figure S3B, Table S7).

**Figure 2:**
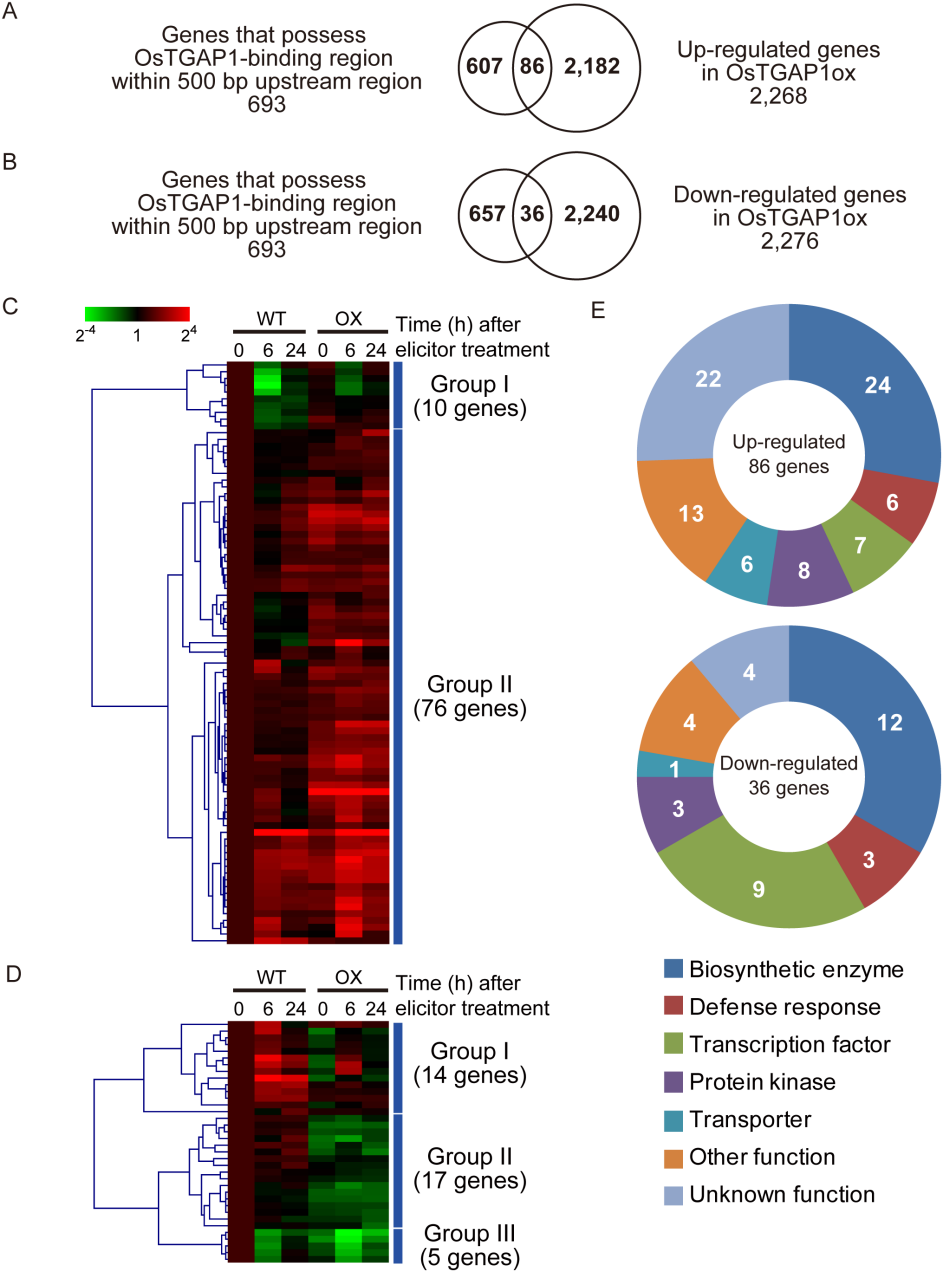
Expression profiles and functional classification of the OsTGAP1 target genes. (A and B) Venn diagrams showing the overlap of genes that possess an OsTGAP1-binding regions within the 400-bp upstream region with the upregulated or downregulated genes in OsTGAP1-overexpressing rice cells. (C and D) Hierarchical clustering of the OsTGAP1 target genes using the cosine correlation and average linkage methods. WT and OX represent wild-type rice cells and OsTGAP1-overexpressing rice cells, respectively. Each column represents the mean of three biological replicates at each time point shown above the heat map. Colours represent induction (red) and repression (green), as indicated by the colour bar. The values of the heat maps are relative to those in the WT cells 0 h after the elicitor treatment. (E) Functional classification of the OsTGAP1 target genes. Categories are as follows: biosynthetic enzyme, defence response, transcription factor, protein kinase, transporter, other function, and unknown function.

To determine the OsTGAP1 target genes, the transcriptome data and ChIP-seq data were compared. Among the 2,763 upregulated genes, 86 (3.1%) possessed OsTGAP1-binding regions in each 400-bp upstream region (Figure 2A, Table S8). Moreover, among the 2,777 downregulated genes, 36 (1.3%) possessed OsTGAP1-binding regions in each 400-bp upstream region (Figure 2B, Table S9). Therefore, these 122 genes were tentatively identified as OsTGAP1 target genes.

Next, the 86 upregulated genes and 36 downregulated genes were subjected to hierarchical clustering. Based on their expression patterns in the WT and OsTGAP1ox cells, the 86 upregulated genes were classified into two groups: group I, whose expression decreased in WT cells after elicitor treatment; and group II, whose expression increased in WT cells after elicitor treatment (Figure 2C). Most of the genes (approximately 88%) were included in group II. The 36 downregulated genes were classified into three groups: group I, whose expression increased in WT cells after elicitor treatment; group II, whose expression was not largely changed in WT cells after elicitor treatment; and group III, whose expression decreased in WT cells after elicitor treatment (Figure 2D). Group I included 14 genes (39%), group II included 17 genes (47%), and group III included 5 genes (14%).

Furthermore, functional classification of these OsTGAP1 target genes was performed. The genes were classified into the following categories according to function: biosynthetic enzyme, defence response, transcription factor, protein kinase, transporter, other function, and unknown function. The results of the functional classification are summarized in Figure 2E. The 86 genes that were upregulated in the OsTGAP1ox cells were classified as follows: biosynthetic enzyme, 24 genes (27.9%); defence response, 6 genes (7.0%); transcription factor, 7 genes (8.1%); protein kinase, 8 genes (9.3%); transporter, 6 genes (7.0%); other function, 13 genes (15.1%); unknown function, 22 genes (25.6%). The 36 genes that were downregulated in the OsTGAP1ox cells were functionally classified as follows: biosynthetic enzyme, 12 genes (33.3%); defence response, 3 genes (8.3%); transcription factor, 9 genes (25%); protein kinase, 3 genes (8.3%); transporter, 1 gene (2.8%); other function, 4 genes (11.1%); unknown function, 4 genes (11.1%).

### OsTGAP1-binding regions in phytoalexin biosynthetic gene clusters

**Figure 3:**
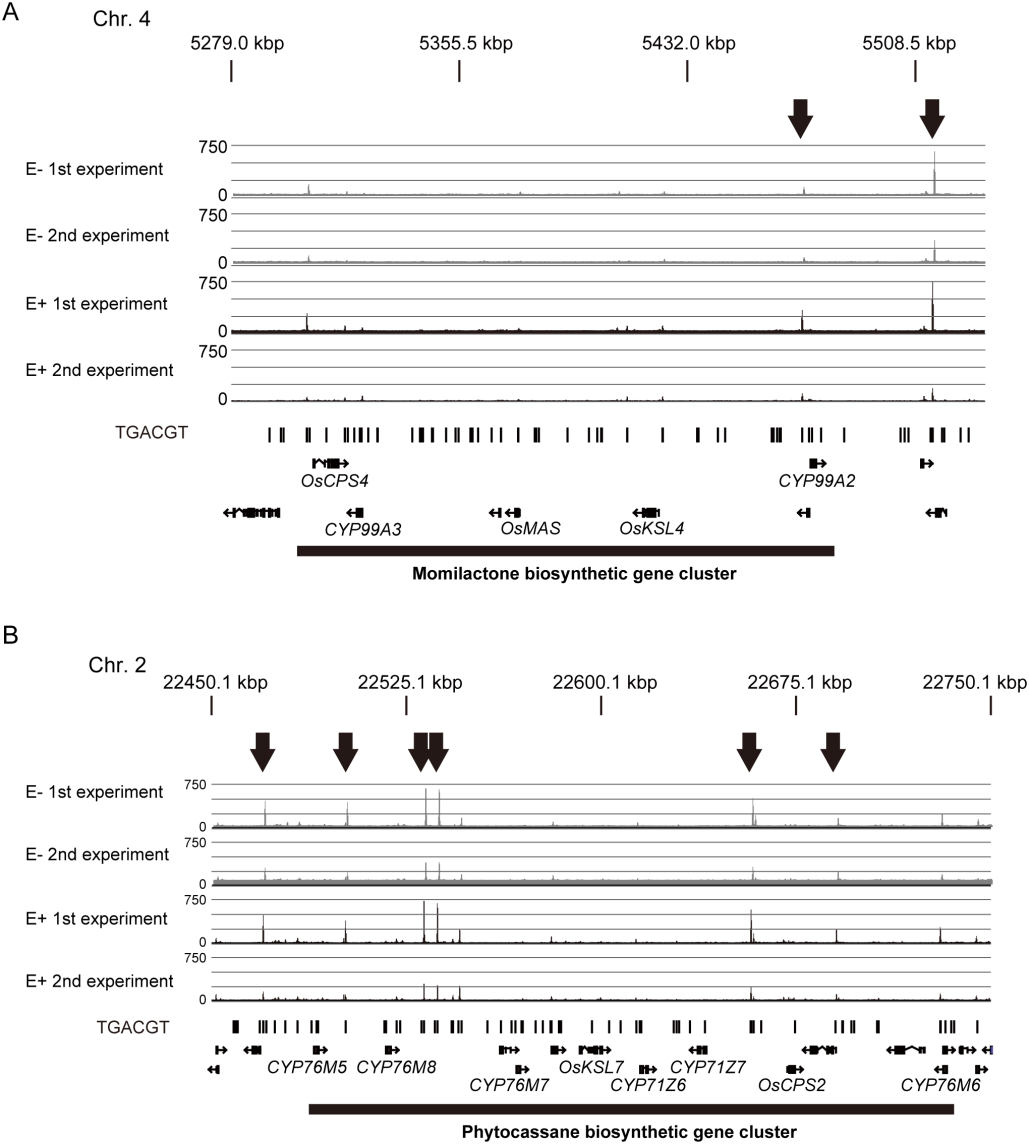
OsTGAP1-binding regions in diterpenoid phytoalexin biosynthetic gene clusters. Mapped ChIP-seq reads in the momilactone (A) and phytocassane biosynthetic gene clusters (B) in untreated (E−, grey) and elicitor-treated (E+, black) conditions, visualized using Partek Genomics Suite. Two biological replicates were performed. Black bars indicate the positions of TGACGT sequences. The genes in these regions are shown in the bottom row.

Although nearly all of the momilactone and phytocassane biosynthetic genes were confirmed to be hyperinductively expressed in the elicitor-stimulated OsTGAP1ox cells through transcriptome analysis (Table S3), none of the momilactone and phytocassane biosynthetic genes in the clusters were identified as OsTGAP1 target genes (Figure S4 and S5). These results suggest that OsTGAP1 does not directly regulate the expression of these genes by binding to their promoter regions. Intriguingly, several OsTGAP1-binding regions were found in intergenic regions among and near the cluster regions (Figure 3). This result raises the possibility that OsTGAP1 positively effects the expression of clustered genes by binding to these regions, although it may also indirectly regulate the expression of these genes via other transcription factors.

### OsTGAP1 binds to the *OsDXS3* promoter via the TGACGT sequence

*OsDXS3*, which encodes 1-deoxy-D-xylulose-5-phosphate synthase (DXS) (EC 2.2.1.7), was included among the OsTGAP1 target genes. The MEP pathway is involved in diterpenoid phytoalexin production in rice [7], and DXS catalyses a key step of this pathway [38]. Therefore, we dissected the transcriptional regulation of *OsDXS3* to understand the regulation of diterpenoid phytoalexin production by OsTGAP1.

**Figure 4:**
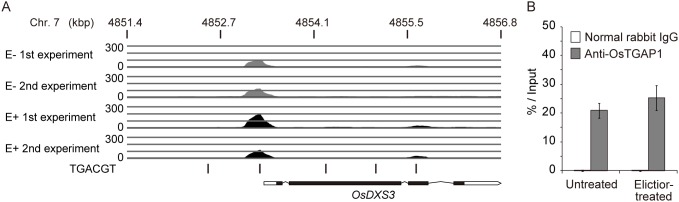
OsTGAP1-binding regions in the *OsDXS3* upstream region. (A) The mapped ChIP-seq reads in untreated (E–, grey) and elicitor-treated (E+, black) conditions were visualized using Partek Genomics Suite. Two biological replicates were performed. Black bars indicate the positions of TGACGT sequences. The gene structure of *OsDXS3* is shown in the bottom row. Open and closed squares indicate untranslated and coding regions, respectively. Lines indicate introns. (B) ChIP-PCR was performed using ChIP DNA immunoprecipitated by the anti-OsTGAP1 antibody and normal rabbit IgG. Values indicate the ratio of the amount of DNA in the ChIP DNA to the amount in the ‘Input’ control (n = 3); bars indicate the standard deviation of the mean.

**Figure 5:**
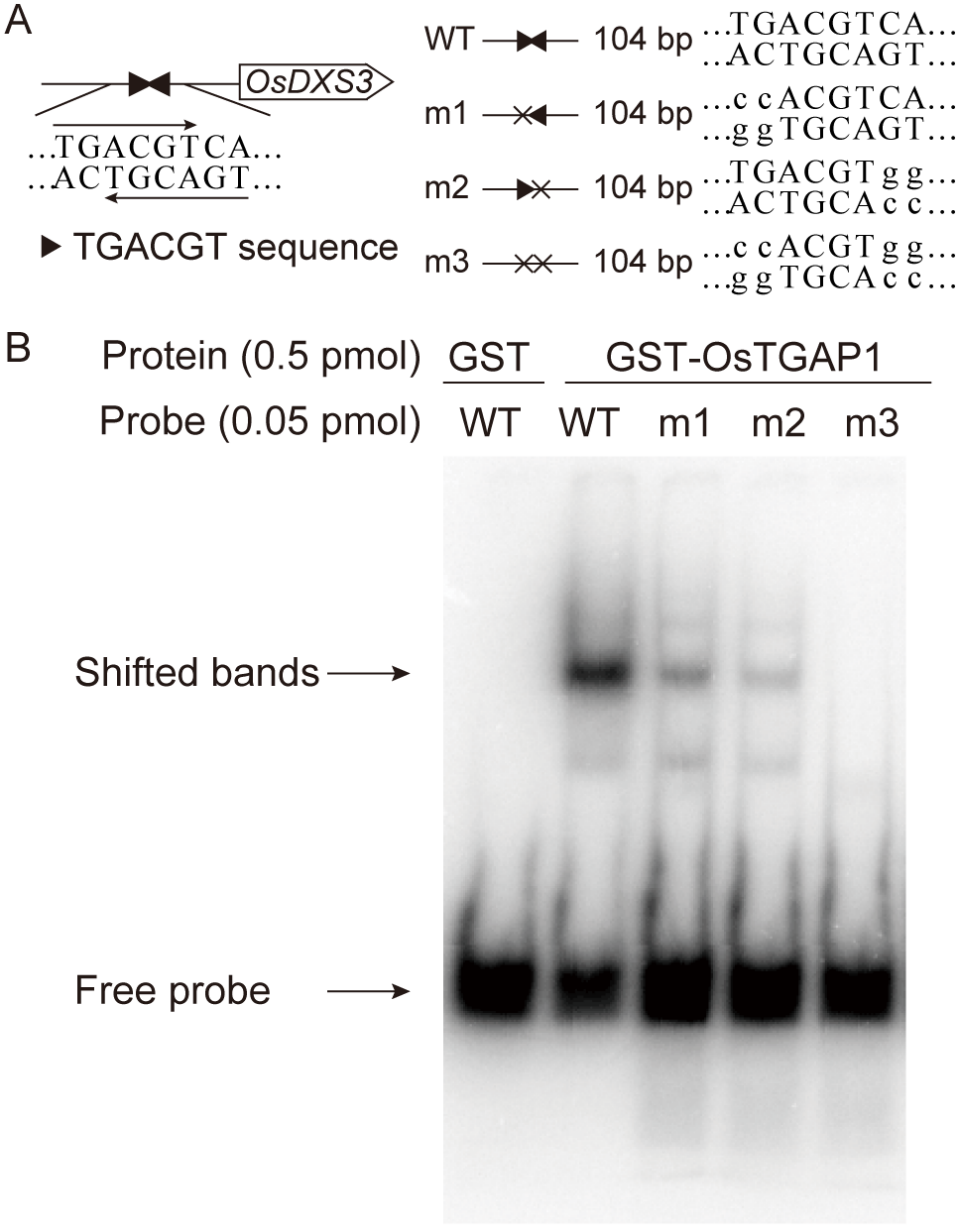
Gel mobility shift assay using the *OsDXS3* upstream region. (A) DNA probes used in GMSA. Closed triangles indicate TGACGT sequences. (B) GMSA was performed using purified recombinant GST-fused OsTGAP1 (GST-OsTGAP1) protein and ^32^P-labelled DNA probes containing the TGACGT sequences in the *OsDXS3* promoter. WT: wild-type probe, m1–m3: mutated probes.

OsTGAP1 bound to an approximately 100-bp upstream region of the transcription start site of *OsDXS3*, both with and without elicitation, in the ChIP-seq analysis (Figure 4A). This binding was confirmed by quantitative PCR using ChIP DNA from another biological replicate. The *OsDXS3* upstream region was enriched in the ChIP DNA immunoprecipitated by the anti-OsTGAP1 antibody compared to that immunoprecipitated by the normal rabbit IgG (Figure 4B), indicating that OsTGAP1 binds to the *OsDXS3* promoter. This OsTGAP1-binding region contained two TGACGT sequences (Figure 5A). GMSAs were performed using a DNA probe containing the *OsDXS3* promoter to investigate whether OsTGAP1 recognized these two TGACGT sequences. As shown in Figure 5B, the GST-fused OsTGAP1 (GST-OsTGAP1) recombinant protein could bind the DNA probe containing the *OsDXS3* promoter. When either TGACGT sequence in this region was mutated (m1 and m2), the binding of GST-OsTGAP1 to the DNA probe was weakened. When both TGACGT sequences were mutated (m3), GST-OsTGAP1 no longer bound to the DNA probe (Figure 5B). Taken together, these results indicate that OsTGAP1 binds to the *OsDXS3* promoter via two TGACGT sequences.

### OsTGAP1 directly regulates *OsDXS3* expression

**Figure 6:**
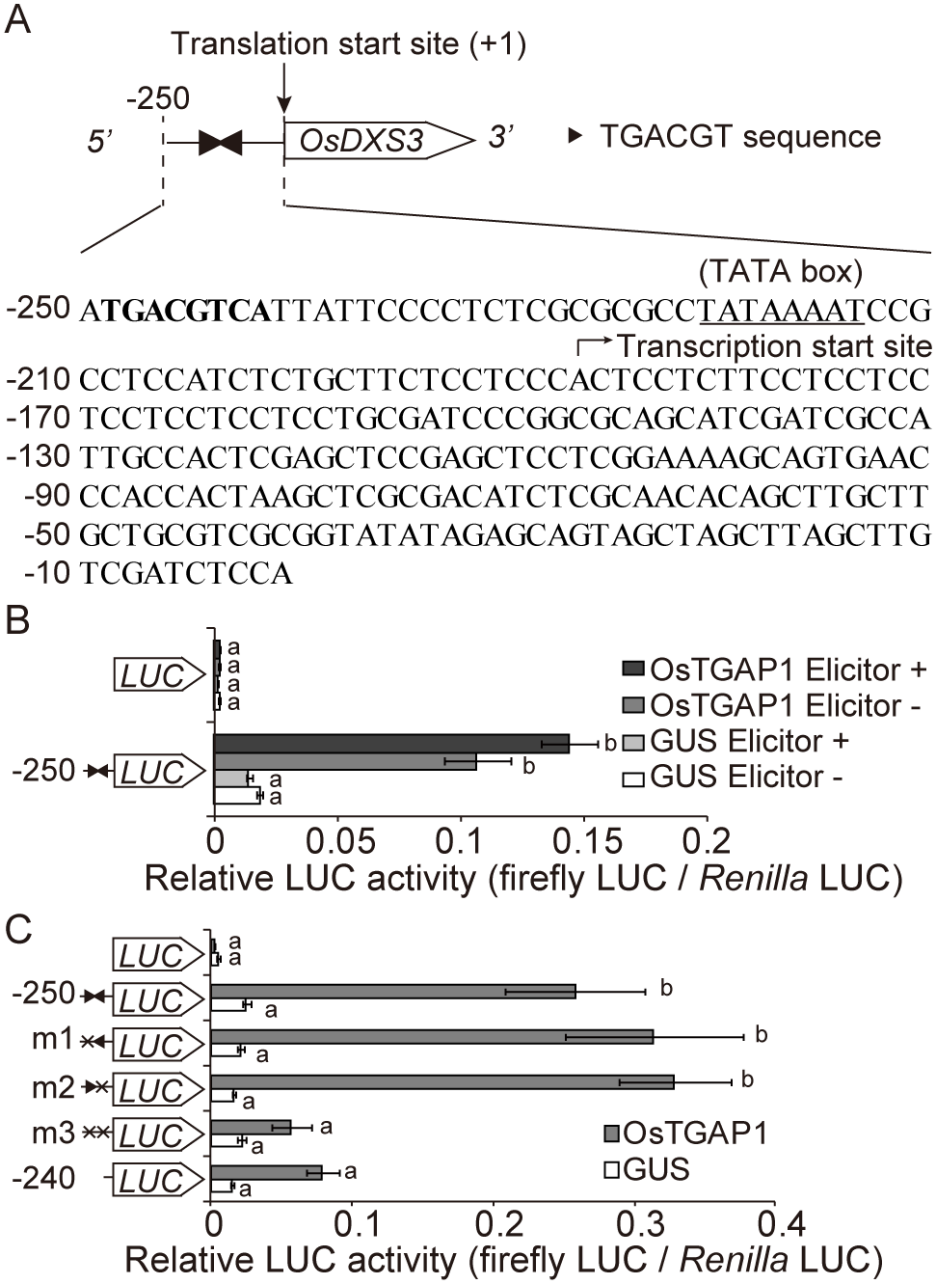
Transactivation assay using a 250-bp upstream region of *OsDXS3.* (A) Nucleotide sequence of the 250-bp upstream region of the ATG translation start site of *OsDXS3*. Closed triangles indicate TGACGT sequences. The TGACGT sequences in the nucleotide sequence are also indicated by bold characters. The putative TATA box is underlined. Transcription start sites on RAP-DB are indicated by black arrows. (B) Transactivation assay using a 250-bp fragment of the *OsDXS3* promoter with GUS or OsTGAP1 effector plasmid (0.1 µg per bombardment). Values indicate the relative luciferase (LUC) activities (firefly LUC/*Renilla* LUC) after 24 h incubation of rice cells with or without chitin elicitor treatment (n = 3); bars indicate the standard error of the mean. Statistically different data groups are indicated by different letters (*P*<0.01 by one-way ANOVA with a Tukey’s *post hoc* test). (C) Transactivation assay using mutated or deleted *OsDXS3* promoters with GUS or OsTGAP1 effector plasmids (0.1 µg per bombardment). Values indicate the relative LUC activities (firefly LUC/*Renilla* LUC) after 24 h incubation of rice cells without chitin elicitor treatment (n = 12); bars indicate the standard error of the mean. Statistically different data groups are indicated by different letters (*P*<0.01 by one-way ANOVA with a Tukey’s post *hoc test*).

To further investigate whether the two TGACGT sequences on the *OsDXS3* promoter are involved in the transcriptional regulation of *OsDXS3*, a transactivation assay was performed. Fragment of 250 bp upstream from the ATG translation start site of *OsDXS3* was cloned and fused to the *FLUC* gene to produce a reporter plasmid (Figure 6A). Effector plasmids were constructed that contained a *GUS* or *OsTGAP1* gene under the control of the maize ubiquitin promoter. The reporter plasmid and either the *GUS* or *OsTGAP1* effector plasmid were delivered into cultured rice cells, along with an internal control plasmid that contained the *RLUC* gene under the control of the CaMV *35S* promoter, by particle bombardment. The LUC activities were quantified and calculated as described in the ‘Materials and Methods’.

Rice cells with the reporter and *OsTGAP1* effector plasmids showed 7-fold higher LUC activity than those with the reporter and *GUS* effector plasmids (Figure 6B). Because *OsDXS3* mRNA level is increased by elicitor treatment, LUC activities after elicitor treatment were also measured. Although the enhancement of LUC activity by OsTGAP1 was detected, no change to LUC activity was observed following elicitor treatment (Figure 6B). These results suggest that a 250-bp fragment of the *OsDXS3* promoter contains elements contributing to the OsTGAP1-dependent induction of *OsDXS3* expression but not to the gene’s responsiveness to the elicitor. A transactivation assay was performed using a fragment of 2 kbp upstream from the ATG translation start site of *OsDXS3*. Rice cells with the reporter plasmid containing a 2-kbp fragment of the *OsDXS3* promoter and the *OsTGAP1* effector plasmid showed higher LUC activity than those with *GUS* effector plasmid. However, LUC activity showed no change under elicitor treatment, as was similar to the results observed for the 250-bp fragment of the *OsDXS3* promoter (Figure S6).

Next, a series of three mutants of the 250-bp fragment of the *OsDXS3* promoter fused to *FLUC* (m1, m2, and m3) were constructed. In m1 and m2, one of the two TGACGT sequences was mutated, while in m3, both TGACGT sequences were mutated. A *FLUC* reporter plasmid containing a 240-bp fragment of the *OsDXS3* promoter was also constructed, and this reporter plasmid did not contain any TGACGT sequences. This series of reporter plasmids was introduced to the cultured rice cells along with either the *GUS* or *OsTGAP1* effector plasmids, and the LUC activities were measured after incubation. The rice cells with m1 or m2 and the *OsTGAP1* effector plasmid exhibited almost the same level of LUC activity as those with the 250-bp promoter construct. However, rice cells with m3 or the 240-bp promoter construct showed significantly lower LUC activity compared to those with the 250-bp promoter construct (Figure 6C). These results indicate that the two TGACGT sequences on the *OsDXS3* promoter are involved in the OsTGAP1-dependent induction of *OsDXS3* expression. Taken together with the fact that OsTGAP1 binds to the *OsDXS3* promoter via these two TGACGT sequences, the results strongly suggest that OsTGAP1 directly regulates *OsDXS3* expression.

## Discussion

### Genome-wide analysis of OsTGAP1 target genes

In this study, ChIP-seq and transcriptome analyses were performed to identify the OsTGAP1 target genes. From the ChIP-seq analysis, the TGACGT sequence was found to be an overrepresented motif in the OsTGAP1-binding regions (Figure 1C, Figure S2). However, there also be the OsTGAP1-binding regions which did not contain any TGACGT sequences (Table S3, Table S4), suggesting that OsTGAP1 may bind to these regions through other motifs. OsTGAP1 may also interact with a DNA-binding protein that recognizes other motifs. However, known binding motifs of other transcription factors were not found in this study (Figure S2). To investigate how OsTGAP1 binds to these regions, a more detailed functional analysis of OsTGAP1 using a biochemical approach is required.

The locations of the OsTGAP1-binding regions assigned to the upstream region were found to be concentrated within 400 bp of the transcription start sites (Figure 1B), suggesting that OsTGAP1 binds near the transcription start sites and regulates the expression of downstream genes. Therefore, this study focussed on those genes that possessed an OsTGAP1-binding region within 400 bp of the upstream region and identified the OsTGAP1 target genes through comparisons to transcriptome data. A total of 86 upregulated genes, along with 36 downregulated genes, were identified in OsTGAP1ox cells (Table S8, Table S9). These target genes were classified based on their expression patterns. Most of the 86 upregulated genes (group II) exhibited an elicitor-induced expression pattern in WT cells. Moreover, this elicitor-induced expression was enhanced in OsTGAP1ox cells (Figure 2C). In light of a previous report that OsTGAP1 shows transactivational activity [17], OsTGAP1 may function to enhance the expression of these genes, including *OsDXS3*. However, the regulatory mechanisms of the 36 genes that were downregulated in OsTGAP1ox cells remain unclear. As these 36 genes could be classified into three groups based on their expression patterns (Figure 2D), each group may be regulated by OsTGAP1 in a different manner. Recent studies have reported that several transcription factors in plants act as both activators and repressors [39], [40]. These transcription factors are thought to interact with coactivators and corepressors, thereby altering these functions. OsTGAP1 may interact with as yet unknown corepressors to suppress the expression of these genes.

A substantial number of the OsTGAP1 target genes encode enzymes of biosynthetic pathways other than terpenoid biosynthesis, such as flavonoid biosynthesis and lipid metabolism (Figure 2E, Table S8, Table S9). This fact suggests that OsTGAP1 is involved not only in the regulation of diterpenoid phytoalexin production but also in the regulation of other biosynthetic processes. In addition, the 86 genes that were upregulated in OsTGAP1ox cells include several defence-related genes, such as glucanase (Os07g0168600) and cystatin (Os01g0803200 and Os01g0915200; Figure 2E, Table S8). Furthermore, *OsPLDbeta1* (Os10g0524400) and *OsWRKY76* (Os09g0417600) were included among the OsTGAP1 target genes that were downregulated in OsTGAP1ox cells (Table S9). As OsPLDbeta1 and OsWRKY76 negatively regulate the rice defence response against *M*. *oryzae* [41], [42], OsTGAP1 may contribute to rice defence responses against pathogens by regulating the expression of these genes. In addition, the OsTGAP1 target genes upregulated in OsTGAP1ox cells included several genes related to plant hormone signalling: *DWARF AND LOW-TILLERING/OsGRAS32* (Os06g0127800), which relates to brassinosteroid signalling [43], and *OsBIF2* (Os12g0614600), which relates to auxin signalling [44] (Table S8). These results raise the possibility that OsTGAP1 impacts the signalling of these plant hormones.

### Regulation of MEP pathway genes by OsTGAP1

OsTGAP1 binds to the *OsDXS3* promoter via two TGACGT sequences (Figure 5B), and these two TGACGT sequences are involved in the OsTGAP1-dependent induction of *OsDXS3* expression (Figure 6C). These results strongly suggest that OsTGAP1 directly regulates *OsDXS3* expression. However, a 250-bp fragment and a 2-kbp fragment of the *OsDXS3* promoter including the OsTGAP1-binding region exhibited no responsiveness to the elicitor (Figure 6B, Figure S6), despite the increase in *OsDXS3* mRNA level due to the elicitor treatment [7]. The regulatory mechanism of the elicitor-induced expression of *OsDXS3* requires further explanation.

The rice genome encodes three *DXS* genes: *OsDXS1*, *OsDXS2*, and *OsDXS3* [45]. Among these three *DXS* genes, *OsDXS3* is the only one upregulated by elicitor treatment [17]. This observation implies that *OsDXS3* is responsible for diterpenoid phytoalexin production and that *OsDXS1* and *OsDXS2* are involved in the biosynthesis of terpenoids for primary metabolism. The OsTGAP1 target genes identified in this study included *OsDXS3* but not *OsDXS1* and *OsDXS2*. The expression of *OsDXS1* and *OsDXS2* was also unchanged in OsTGAP1ox cells (Table S10). These results suggest that the transcriptional regulation of the MEP pathway by OsTGAP1 mainly contributes to diterpenoid phytoalexin production and that its contribution to the biosynthesis of terpenoids for primary metabolism is limited.

Of the MEP pathway genes, only *OsDXS3* was included among the OsTGAP1 target genes. Nevertheless, two MEP pathway genes (*OsHDS* and *OsHDR*) were found among the upregulated genes in OsTGAP1ox cells (Table S3). Other MEP pathway genes (*OsDXR*, *OsCMS*, *OsCMK*, and *OsMCS*) were also upregulated by approximately 1.5-fold 24 h after elicitor treatment in the OsTGAP1ox cells compared to their expression after treatment in WT cells, although these genes were eliminated by the threshold of the data analysis (Table S10). These results indicate that the expression of all seven MEP pathway genes is affected by OsTGAP1. However, *OsDXS3* was more strongly upregulated than were the other MEP pathway genes in OsTGAP1ox cells. Therefore, the enhanced expression of MEP pathway genes by OsTGAP1, except that of *OsDXS3*, may be indirect.

### Possible mechanism for the regulation of diterpenoid phytoalexin biosynthetic gene clusters by OsTGAP1

OsTGAP1ox cells exhibit enhanced expression of all momilactone biosynthetic genes and the phytocassane biosynthetic gene *OsKSL7* [17]. In this study, other phytocassane biosynthetic genes in the cluster (*OsCPS2*, *CYP76M5-8*, and *CYP71Z6*) were found to be upregulated in the OsTGAP1ox cells (Table S3), suggesting that OsTGAP1 functions in the transcriptional regulation of the two diterpenoid phytoalexin biosynthetic gene clusters. However, a simple model in which OsTGAP1 directly regulates the expression of these biosynthetic genes through binding to each promoter region followed by transactivation was not supported by the ChIP-seq analysis (Figure 3, Figure S4 and Figure S5).

Several transcription factor genes were found among the candidate OsTGAP1 target genes, including *OsWRKY76* (Figure 2E, Table S8, Table S9). Recently, *OsWRKY76* has been reported to negatively regulate diterpenoid phytoalexin biosynthetic genes [42]. Therefore, the downregulation of *OsWRKY76* expression may be among the mechanisms explaining the enhanced expression of diterpenoid phytoalexin biosynthetic genes in OsTGAP1ox cells.

Another hypothesis is that the binding of OsTGAP1 to the intergenic regions of the gene clusters plays a particular role in the transcriptional regulation of diterpenoid phytoalexin biosynthetic genes. In this study, at least 40% of the OsTGAP1-binding regions were located within intergenic regions (Figure 1A). This proportion is larger than those observed for the other plant transcription factors (HY5, PIL5, AGL15, and BZR1) whose genome-wide binding regions have been previously reported [33]–[36]. However, in the case of FHY3, which is a component of phytochrome A signalling, approximately 40% of FHY3-binding regions are located in intergenic regions, and a large portion of these intergenic binding regions are localized in the centromeric regions [46]. FHY3 binds to the promoter regions of its target genes, thereby regulating phytochrome A signalling and the circadian clock. FHY3 also binds to the centromeric repeats, suggesting that FHY3 has a function beyond regulating the expression of target genes via binding to their promoter regions. Similar to that of FHY3, the binding of OsTGAP1 to intergenic regions may also have unknown but essential functions. The information presented in this study regarding OsTGAP1-binding regions near and in the diterpenoid phytoalexin biosynthetic gene cluster regions will contribute to future research investigating the regulation of these gene clusters by OsTGAP1.

## Supporting Information

Figure S1 Specificity of anti-OsTGAP1 antibody.
(PDF)

Figure S2 The enrichment of each motif in OsTGAP1-binding regions.
(PDF)

Figure S3 Summary of genes whose expression was altered in OsTGAP1-overexpressing rice cells.
(PDF)

Figure S4 OsTGAP1-binding regions around momilactone biosynthetic genes.
(PDF)

Figure S5 OsTGAP1-binding regions around phytocassane biosynthetic genes.
(PDF)

Figure S6 Transactivation assay using the 2-kbp fragment of the *OsDXS3* promoter.
(PDF)

Table S1 Plasmids used in this study.
(DOCX)

Table S2 Primers used in this study.
(DOCX)

Table S3 List of OsTGAP1-binding regions in untreated condition.
(XLSX)

Table S4 List of OsTGAP1-binding regions in elicitor-treated condition.
(XLSX)

Table S5 List of genes that possess OsTGAP1-binding site within 400 bp upstream region.
(XLSX)

Table S6 List of genes whose expression is significantly upregulated in OsTGAP1-overexpressinng rice cells.
(XLSX)

Table S7 List of genes whose expression is significantly downregulated in OsTGAP1-overexpressinng rice cells.
(XLSX)

Table S8 List of OsTGAP1 target genes whose expression is upregulated in OsTGAP1-overexpressinng rice cells.
(XLSX)

Table S9 List of OsTGAP1 target genes whose expression is downregulated in OsTGAP1-overexpressinng rice cells.
(XLSX)

Table S10 Expression profiles of MEP pathway genes from microarray analysis.
(XLSX)
