# Supplementary material for: Identification of Target Genes of the bZIP Transcription Factor OsTGAP1, Whose Overexpression Causes Elicitor-Induced Hyperaccumulation of Diterpenoid Phytoalexins in Rice Cells
Source: PLoS One. 2014 Aug 26;9(8):e105823. doi: 10.1371/journal.pone.0105823 (PMC4144896; doi:10.1371/journal.pone.0105823)
Supplement: Figure S1 — Specificity of anti-OsTGAP1 antibody. (PDF) [file pone.0105823.s001.pdf]

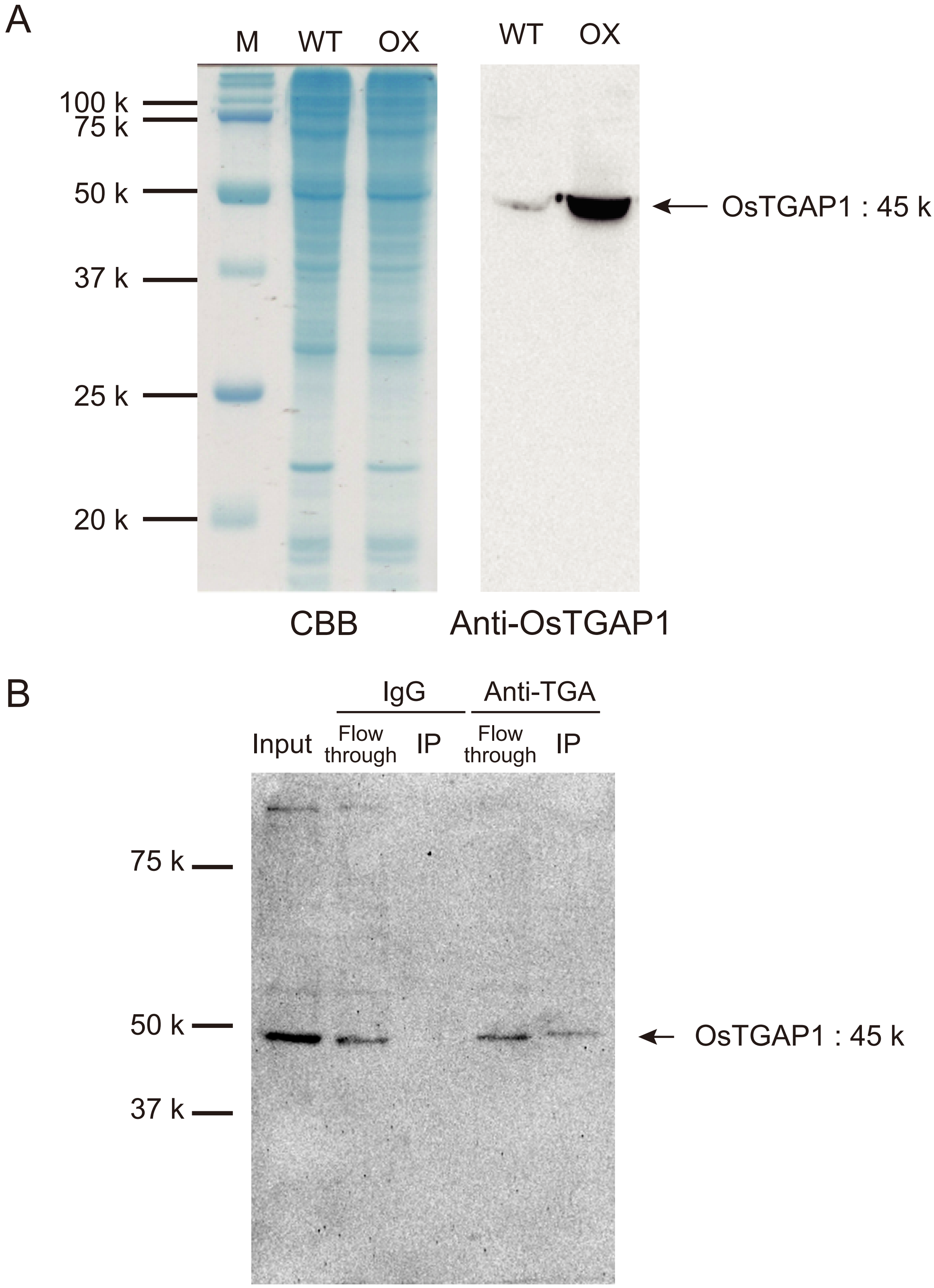

Fig. S1. Specificity of anti-OsTGAP1 antibody. (A) Protein gel blot analysis of nuclear extracts from wild-type rice cells (WT) and OsTGAP1-overexpressing rice cells (OX) using anti-OsTGAP1 antibody. Nuclear extracts from WT and OX were separated by SDS-PAGE and stained with Coomassie Brilliant Blue (CBB) (left panel). Separated nuclear extracts were also blotted on a membrane and probed with anti-OsTGAP1 antibody (right panel). (B) Nuclear extracts from WT were subject to protein gel blot analysis after immunoprecipitation by anti-OsTGAP1 antibody (anti-TGA) and normal rabbit IgG (IgG). Input, 'Input' control; IP, immunoprecipitated protein.
