# Supplementary material for: Identification of Target Genes of the bZIP Transcription Factor OsTGAP1, Whose Overexpression Causes Elicitor-Induced Hyperaccumulation of Diterpenoid Phytoalexins in Rice Cells
Source: PLoS One. 2014 Aug 26;9(8):e105823. doi: 10.1371/journal.pone.0105823 (PMC4144896; doi:10.1371/journal.pone.0105823)
Supplement: Figure S2 — The enrichment of each motif in OsTGAP1-binding regions. (PDF) [file pone.0105823.s002.pdf]

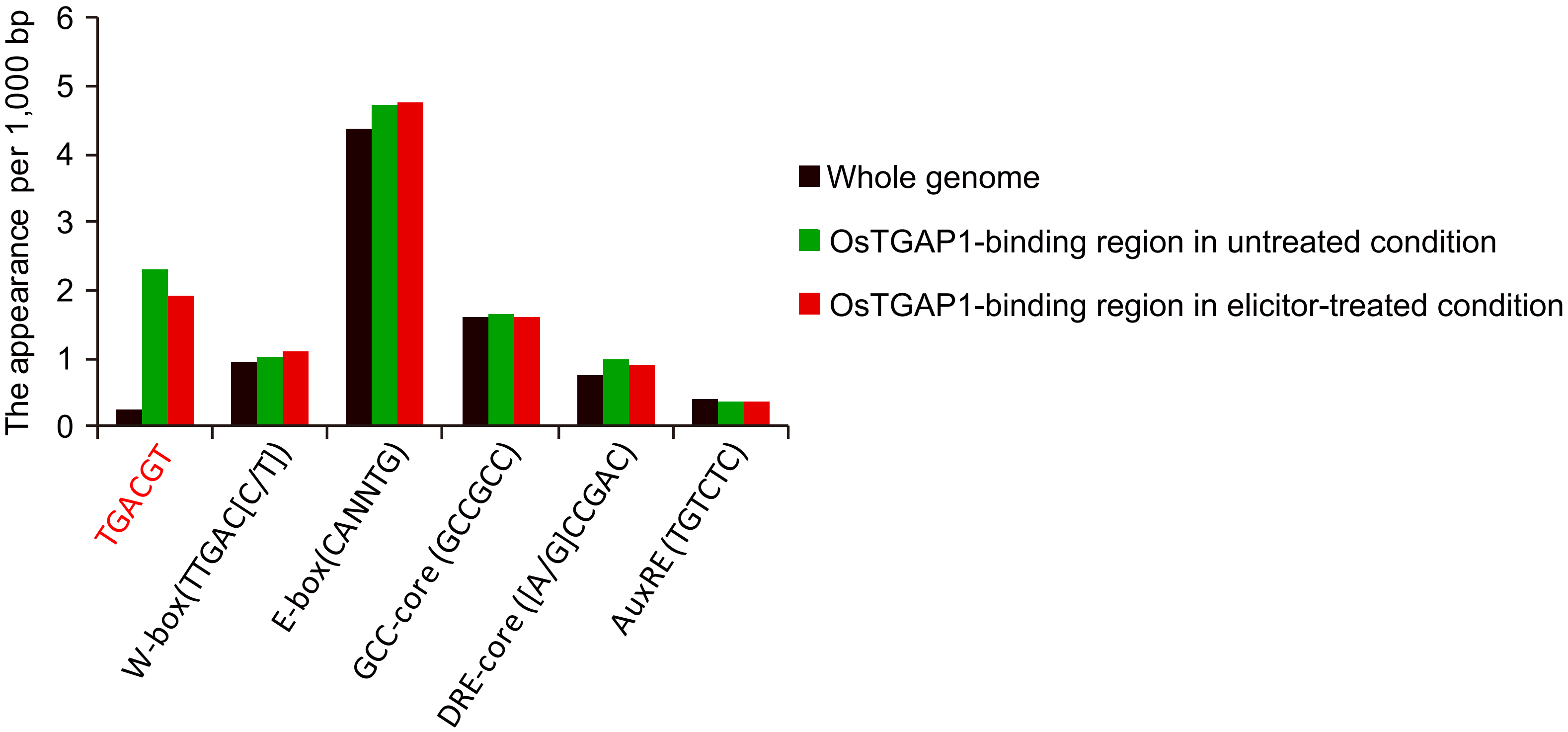

Fig. S2. The enrichment of each motif in OsTGAP1-binding regions. Y-axis represents the appearance of each motif in the whole genome sequence or OsTGAP1-binding regions.
