# Supplementary material for: Identification of Target Genes of the bZIP Transcription Factor OsTGAP1, Whose Overexpression Causes Elicitor-Induced Hyperaccumulation of Diterpenoid Phytoalexins in Rice Cells
Source: PLoS One. 2014 Aug 26;9(8):e105823. doi: 10.1371/journal.pone.0105823 (PMC4144896; doi:10.1371/journal.pone.0105823)
Supplement: Figure S3 — Summary of genes whose expression was altered in OsTGAP1-overexpressing rice cells. (PDF) [file pone.0105823.s003.pdf]

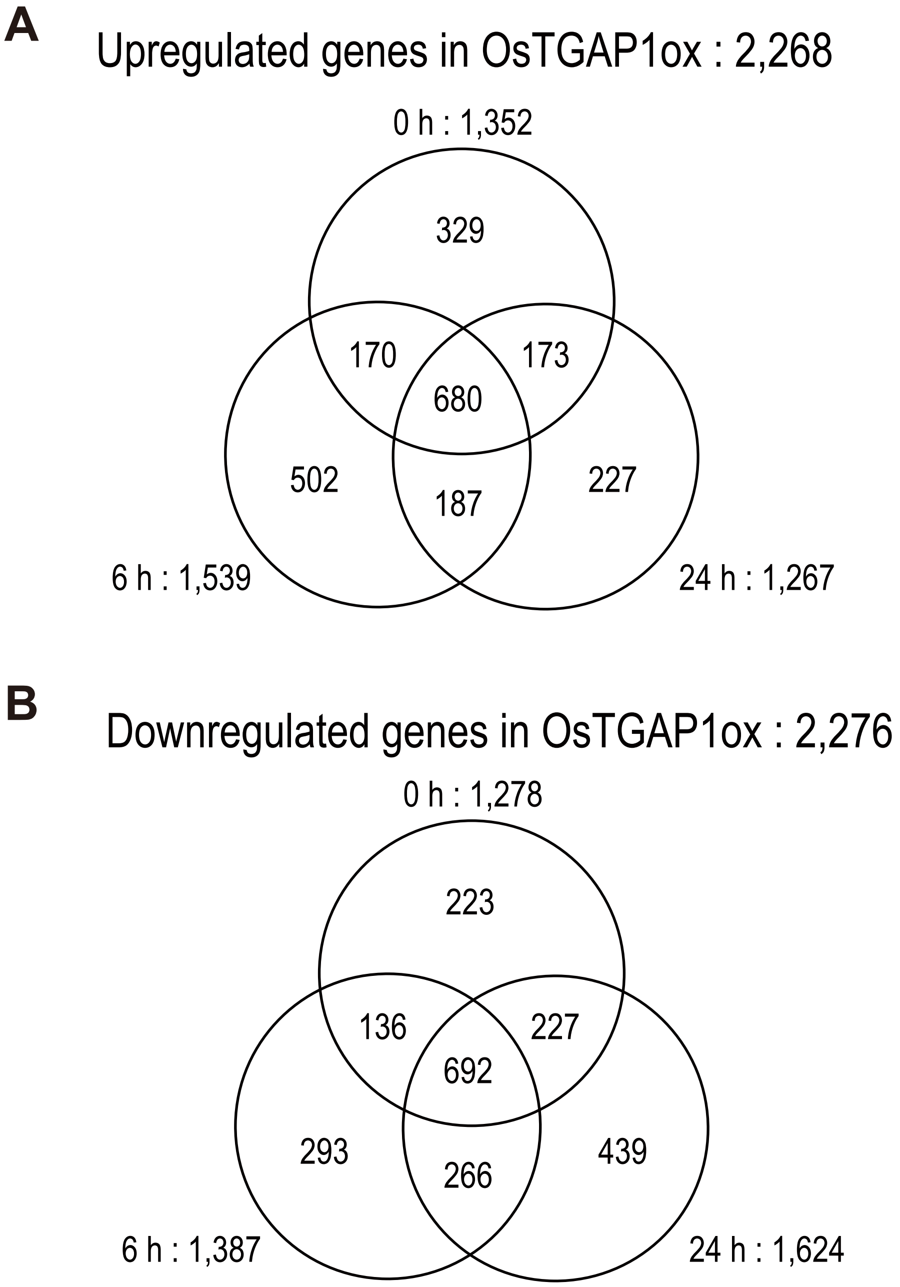

Fig. S3. Summary of genes whose expression was altered in OsTGAP1-overexpressing rice cells compared to wild-type rice cells. The genes whose expressions were changed in OsTGAP1-overexpressing rice cells were analysed using the Agilent rice 44k oligoarray with the one-colour method. Venn diagrams show the number of overlapping and unique genes. Genes that were (A) upregulated or (B) downregulated more than two-fold in OsTGAP1-overexpressing rice cells compared to wild-type cells are shown.
