# Supplementary material for: Identification of Target Genes of the bZIP Transcription Factor OsTGAP1, Whose Overexpression Causes Elicitor-Induced Hyperaccumulation of Diterpenoid Phytoalexins in Rice Cells
Source: PLoS One. 2014 Aug 26;9(8):e105823. doi: 10.1371/journal.pone.0105823 (PMC4144896; doi:10.1371/journal.pone.0105823)
Supplement: Figure S5 — OsTGAP1-binding regions around phytocassane biosynthetic genes. (PDF) [file pone.0105823.s005.pdf]

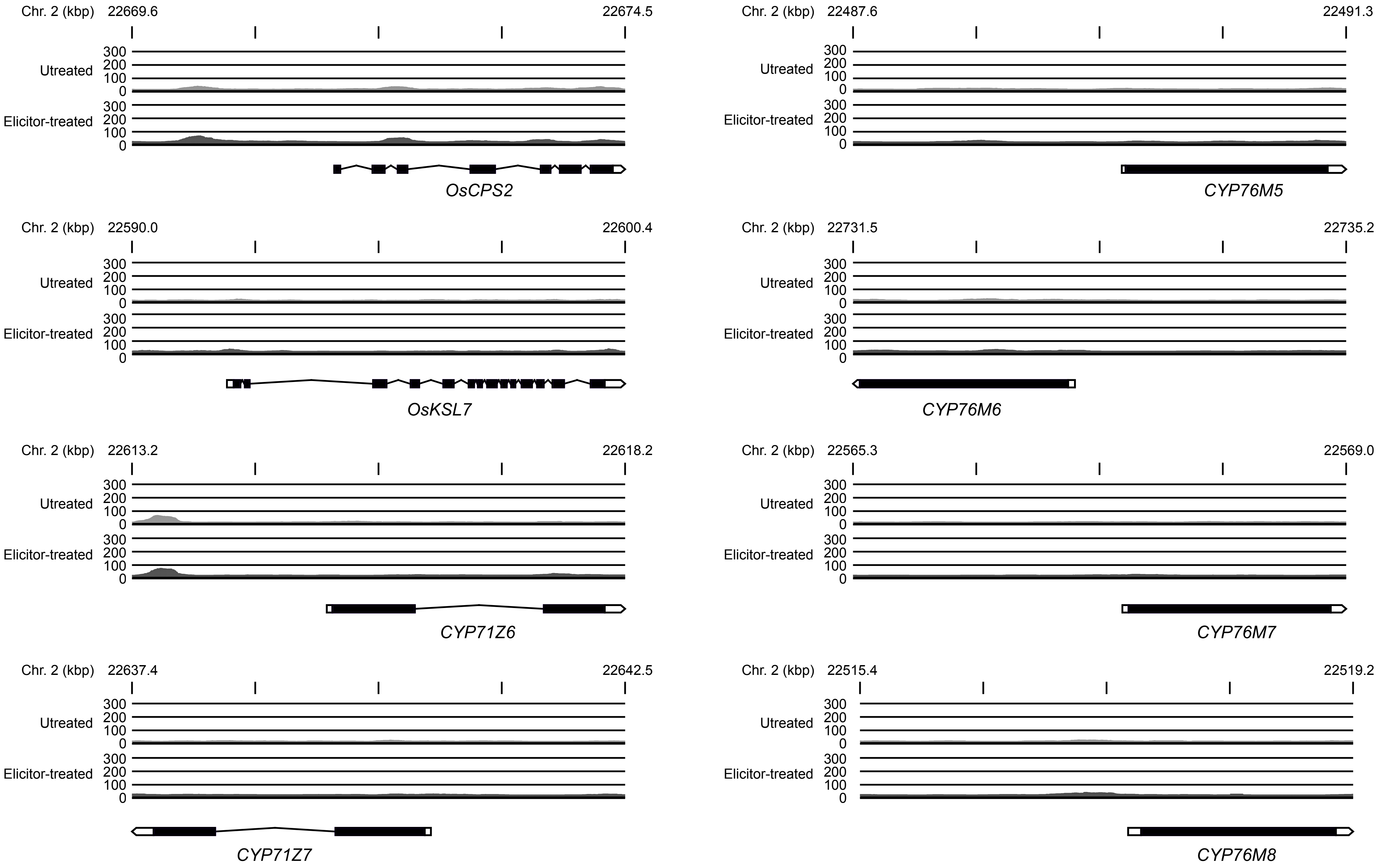

Fig. S5. OsTGAP1-binding regions around phytocassane biosynthetic genes. The mapped ChIP-seq reads in untreated (grey) and elicitor-treated (black) conditions were visualized using Partek Genomics Suite. No binding site was detected in the 2-kbp upstream region of each gene. The gene structure of each phytocassane biosynthetic gene is shown in the bottom row. Open and closed squares indicate untranslated and coding regions, respectively. Lines indicate introns.
