# Supplementary material for: Identification of Target Genes of the bZIP Transcription Factor OsTGAP1, Whose Overexpression Causes Elicitor-Induced Hyperaccumulation of Diterpenoid Phytoalexins in Rice Cells
Source: PLoS One. 2014 Aug 26;9(8):e105823. doi: 10.1371/journal.pone.0105823 (PMC4144896; doi:10.1371/journal.pone.0105823)
Supplement: Figure S6 — Transactivation assay using the 2-kbp fragment of the OsDXS3 promoter. (PDF) [file pone.0105823.s006.pdf]

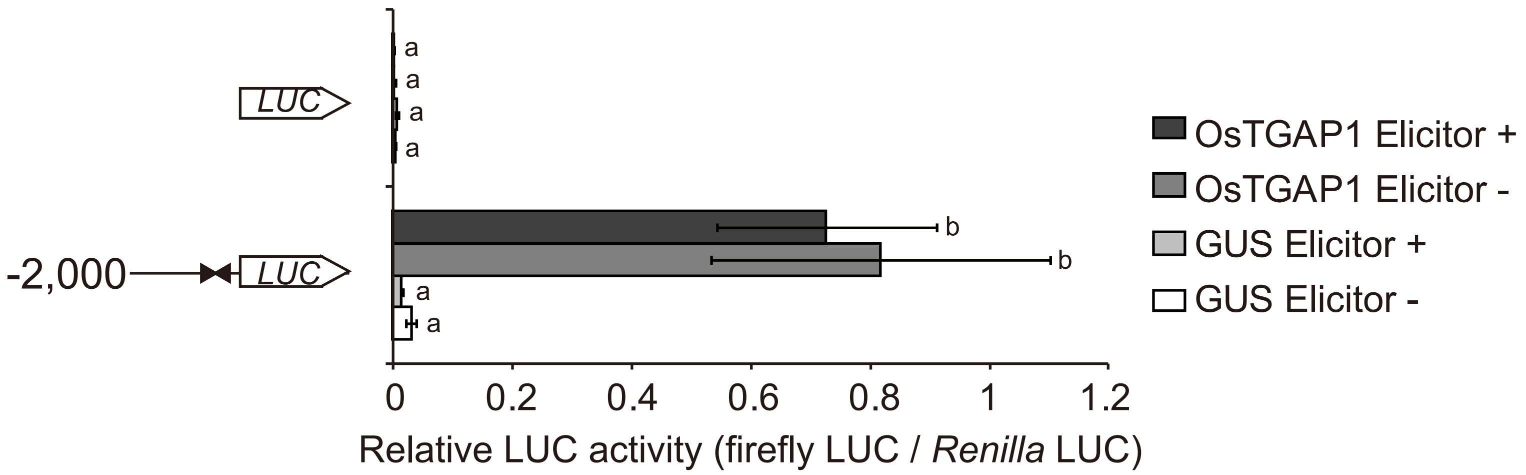

Fig. S6. Transactivation assay using the 2-kbp fragment of the *OsDXS3* promoter. *GUS* or *OsTGAP1* effector plasmid (1 μg per bombardment) was used. Values indicate the relative luciferase (LUC) activities (firefly LUC / *Renilla* LUC) after 24 h incubation of rice cells with or without chitin elicitor treatment (n = 4); bars indicate the standard error of the mean. Statistically different data groups are indicated by different letters ($P < 0.01$ by one-way ANOVA with a Tukey post hoc test).
