# Supplementary material for: Identification of Target Genes of the bZIP Transcription Factor OsTGAP1, Whose Overexpression Causes Elicitor-Induced Hyperaccumulation of Diterpenoid Phytoalexins in Rice Cells
Source: PLoS One. 2014 Aug 26;9(8):e105823. doi: 10.1371/journal.pone.0105823 (PMC4144896; doi:10.1371/journal.pone.0105823)
Supplement: Table S1 — Plasmids used in this study. (DOCX) [file pone.0105823.s007.docx]

**Table S1. Plasmids used in this study**

| **Plasmid** | **Plasmid design and markers** | **Reference** |
| --- | --- | --- |
| pANDA | Hm^R^, Km^R^, maize *ubiquitin* promoter, *NOS* terminator, Gateway vector | [[1](#_ENREF_1)] |
| pDONR^TM^P4-P1R | Km^R^ | Invitrogen |
| pDONR-UBQp | Km^R^, pDONR^TM^P4-P1R containing maize *ubiquitin* promoter | This study |
| R4pGWB501 | Hm^R^, Km^R^, Spc^R^, *NOS* terminator, Gateway vector | [[2](#_ENREF_2)] |
| pENTR-TGA | Km^R^, pENTR/D-TOPO (Invitrogen) containing *OsTGAP1* ORF | [[3](#_ENREF_3)] |
| R4pGWB-UBQp-TGA | Hm^R^, Km^R^, Spc^R^, R4pGWB501 containing maize *ubiquitin* promoter and *OsTGAP1* ORF | This study |
| pGL3 basic | Amp^R^, firefly luciferase gene | Promega |
| pGL3-DXS3p-2k | Amp^R^, pGL3 basic containing 2 kbp upstream region of *OsDXS3* | This study |
| pGL3-DXS3p-2k-m1 | Amp^R^, pGL3 basic containing 2 kbp upstream region of *OsDXS3*, one TGACGT sequence is mutated | This study |
| pGL3-DXS3p-2k-m2 | Amp^R^, pGL3 basic containing 2 kbp upstream region of *OsDXS3*, one TGACGT sequence is mutated | This study |
| pGL3-DXS3p-2k-m3 | Amp^R^, pGL3 basic containing 2 kbp upstream region of *OsDXS3*, two TGACGT sequences are mutated | This study |
| pGL3-DXS3p-250 | Amp^R^, pGL3 basic containing 250 bp upstream region of *OsDXS3* | This study |
| pGL3-DXS3p-250-m1 | Amp^R^, pGL3 basic containing 250 bp upstream region of *OsDXS3*, one TGACGT sequence is mutated | This study |
| pGL3-DXS3p-250-m2 | Amp^R^, pGL3 basic containing 250 bp upstream region of *OsDXS3*, one TGACGT sequence is mutated | This study |
| pGL3-DXS3p-250-m3 | Amp^R^, pGL3 basic containing 250 bp upstream region of *OsDXS3*, two TGACGT sequences are mutated | This study |
| pGL3-DXS3p-240 | Amp^R^, pGL3 basic containing 240 bp upstream region of *OsDXS3* | This study |
| pGEX-6p2 | Amp^R^ | GE healthcare |
| pDEST15-TGA | Amp^R^, pDEST15 containing OsTGAP1 ORF | [[3](#_ENREF_3)] |
| pUbi_RfA_Tnos | Amp^R^, maize *ubiquitin* promoter, *NOS* terminator, Gateway vector | [[4](#_ENREF_4)] |
| pUbi_TGA_Tnos | Amp^R^, pUbi_RfA_Tnos containing *OsTGAP1* ORF | This study |
| pUbi_GUS_Tnos | Amp^R^, pUbi_RfA_Tnos containing *GUS* gene | [[4](#_ENREF_4)] |
| pPTRL | Amp^R^, CaMV *35S* promoter-Renilla *LUC* | [[5](#_ENREF_5)] |

1. Miki D, Shimamoto K (2004) Simple RNAi Vectors for Stable and Transient Suppression of Gene Function in Rice. Plant Cell Physiol 45: 490-495.

2. Nakagawa T, Nakamura S, Tanaka K, Kawamukai M, Suzuki T, et al. (2008) Development of R4 Gateway Binary Vectors (R4pGWB) Enabling High-Throughput Promoter Swapping for Plant Research. Biosci Biotechnol Biochem 72: 624-629.

3. Okada A, Okada K, Miyamoto K, Koga J, Shibuya N, et al. (2009) OsTGAP1, a bZIP transcription factor, coordinately regulates the inductive production of diterpenoid phytoalexins in rice. J Biol Chem 284: 26510-26518.

4. Chujo T, Miyamoto K, Ogawa S, Masuda Y, Shimizu T, et al. (2014) Overexpression of Phosphomimic Mutated OsWRKY53 Leads to Enhanced Blast Resistance in Rice. PLoS ONE 9: e98737.

5. Ohta M, Ohme-Takagi M, Shinshi H (2000) Three ethylene-responsive transcription factors in tobacco with distinct transactivation functions. The Plant J 22: 29-38.
