# Supplementary material for: Identification of Target Genes of the bZIP Transcription Factor OsTGAP1, Whose Overexpression Causes Elicitor-Induced Hyperaccumulation of Diterpenoid Phytoalexins in Rice Cells
Source: PLoS One. 2014 Aug 26;9(8):e105823. doi: 10.1371/journal.pone.0105823 (PMC4144896; doi:10.1371/journal.pone.0105823)
Supplement: Table S2 — Primers used in this study. (DOCX) [file pone.0105823.s008.docx]

**Table S2. Primers used in this study**

| Primer | Sequence |
| --- | --- |
| UBQp attB4　F | 5’-GGGGACAACTTTGTATAGAAAAGTTGTGGGCTGCAGTGCAGCGTGAC-3’ |
| UBQp attB1　R | 5’-GGGGACTGCTTTTTTGTACAAACTTGTGCAGAAGTAACACCAAACAACAG-3’ |
| DXS3p 2k F | 5’-GGGGGTACCTATATATGTGTAAAGTTGGATCTTG-3’ |
| DXS3p R | 5’-GGGAAGCTTTGGAGATCGACAAGCTAAGC-3’ |
| DXS3p m1 F | 5’-GTTTTATTTATTTACCACGTCATTATTCCCCTCT-3’ |
| DXS3p m1 R | 5’-AGAGGGGAATAATGACGTGGTAAATAAATAAAAC-3’ |
| DXS3p m2 F | 5’-GTTTTATTTATTTATGACGTGGTTATTCCCCTCT-3’ |
| DXS3p m2 R | 5’-AGAGGGGAATAACCACGTCATAAATAAATAAAAC-3’ |
| DXS3p m3 F | 5’-GTTTTATTTATTTACCACGTGGTTATTCCCCTCT-3’ |
| DXS3p m3 R | 5’- AGAGGGGAATAACCACGTGGTAAATAAATAAAAC-3’ |
| DXS3p 250 F | 5’-AGATCTATGACGTCATTATTCCCCTC-3’ |
| DXS3p 240 F | 5’-AGATCTTATTCCCCTCTCGCGCGCC-3’ |
| DXS3p 250 m1 F | 5’-AGATCTACCACGTCATTATTCCCCTC-3’ |
| DXS3p 250 m2 F | 5’-AGATCTATGACGTGGTTATTCCCCTC-3’ |
| DXS3p 250 m3 F | 5’-AGATCTACCACGTGGTTATTCCCCTC-3’ |
| DXS3p TGACGT F | 5’-CTCCCGCTAAAACTTGCTTG-3’ |
| DXS3p TGACGT R | 5’-TGGGAGGAGAAGCAGAGAATG-3’ |

Restriction enzyme sites are indicated by underline. Mutated sequence is indicated in red letters.
